# Design and fabrication of novel microfluidic-based droplets for drug screening on a chronic myeloid leukemia cell line

**DOI:** 10.1371/journal.pone.0315803

**Published:** 2025-01-15

**Authors:** Niloofar Jaafari, Amir Asri Kojabad, Rima Manafi Shabestari, Majid Safa

**Affiliations:** 1 Department of Hematology and Blood Banking, Faculty of Allied Medicine, Iran University of Medical Science, Tehran, Iran; 2 Department of Pharmacology and Chemical Biology, University of Pittsburgh School of Medicine, Pittsburgh, Pennsylvania, United States of America; Duke University Medical Center: Duke University Hospital, UNITED STATES OF AMERICA

## Abstract

**Background:**

The challenges associated with traditional drug screening, such as high costs and long screening times, have led to an increase in the use of single-cell isolation technologies. Small sample volumes are required for high-throughput, cell-based assays to reduce assay costs and enable rapid sample processing. Using microfluidic chips, single-cell analysis can be conducted more effectively, requiring fewer reagents and maintaining biocompatibility. Due to the chip’s ability to manipulate small volumes of fluid, high-throughput screening assays can be developed that are both miniaturized and automated. In the present study, we employ microfluidic chips for drug screening in chronic myeloid leukemia. This study aimed to establish a robust methodology integrating diverse assays, providing a holistic understanding of drug response.

**Material and methods:**

Herein, we have used a chronic myeloid leukemia derived cell line (K562) for drug screening with an innovative microfluidic-based drug screening approach to investigate the efficacy of imatinib in K562 cells. Cell viability was assessed using MTT assay. Apoptosis was measured using Annexin/PI staining by flow cytometry.

**Results:**

Significant increased apoptosis was seen in K562 cells treated with imatinib in the microfluidic device compared to cells treated with imatinib in 24- and 96-well plates. Moreover, in the microfluidic chip, drug screening time was reduced from 48 hours to 24 hours.

**Conclusion:**

Compared to traditional approaches, microfluidic-based drug screening efficiently evaluates the efficacy of imatinib in K562 cells. This approach is promising for drug discovery and treatment optimization, as it increases sensitivity and streamlines the screening process.

## 1. Introduction

Traditionally, drug testing is performed in multiwell plates, which is expensive, time-consuming, and requires large quantities of reagents [1, 2]. For overcoming these limitations and gaining a deeper understanding of cell heterogeneity, disease diagnosis, and cancer biology, single-cell isolation technologies have become indispensable [3]. However, due to the small size and low concentration of biomolecules, analyzing single cells can be challenging [4]. For the study of cell population heterogeneity, microfluidic devices have proven to be particularly effective, offering high throughput, automatic processing, low contamination risk, simple control, and biocompatibility [5–7].

Using microfluidic chips facilitates drug screening and reagent mixing, enabling the generation of varying concentrations without manual pipetting. The use of microfluidic chips has proven valuable in determining dose-dependent cellular responses at different concentrations, a crucial aspect of drug screening and treatment optimization [6, 8, 9]. The large-scale evaluation and quantitative of the optimal concentrations and toxicity of diverse drugs further enhances throughput while reducing experimental expenses [1]. Current microfluidic chip applications commonly involve the administration of two or more drug doses. To allow for the evaluation of individual drugs on heterogeneous tumor cell populations, multifunctional integrated microfluidic platforms have been developed, enabling precise single-cell drug screening [10]. One promising approach is droplet microfluidics, which allows for the high-throughput generation of monodispersed, nanoliter-sized droplets as individual reaction chambers. These droplets, produced at kilohertz rates, facilitate efficient and rapid drug compound screening through oil-in-water or water-in-oil emulsions. The droplet volumes are adjustable, ranging from picolitres to nanoliters, through modifications to the system’s parameters. This technology improves reagent mixing due to the large surface-to-volume ratio and 3D flow structure of the droplets while minimizing processing steps. Moreover, droplet microfluidics reduces screening time by accelerating drug-cell interactions and optimizing fluid flow, which is the focus of this study to demonstrate its efficiency as an alternative to traditional methods [11, 12].

As mentioned above, using droplet microfluidics in drug screening offers numerous advantages, particularly in studying drug responses [13, 14]. This is particularly relevant in chronic myeloid leukemia (CML), a clonal disorder characterized by the Philadelphia chromosome. Although there are some concerns related to resistance and toxicity of the imatinib drug, it remains an effective choice of treatment [1, 15].

In our study, to overcome the aforementioned challenges, single-cell capture array and droplet microfluidic chip have been combined to create a single-cell microfluidic drug screening platform. In this study, we selected the K562 and NB4 leukemia cell lines to investigate the efficacy of our microfluidic platform in drug screening for hematological malignancies and introduce an alternative to time-consuming traditional drug screening methods. K562, a chronic myeloid leukemia (CML) cell line, is commonly used due to its sensitivity to imatinib, a tyrosine kinase inhibitor (TKI) widely used as a first-line treatment for CML. The use of K562 allows us to model the therapeutic effects of imatinib, making it ideal for evaluating the performance of new drug screening technologies. On the other hand, NB4 is an acute promyelocytic leukemia (APL) cell line known for its resistance to imatinib. This contrast provides a robust system for evaluating drug responses across different leukemic contexts, particularly in comparing drug-sensitive and drug-resistant phenotypes. By selecting these two suspended cell lines, we aim to demonstrate the versatility of our platform in screening drugs for hematological malignancies, offering insights into its broader applicability. This study employs a polydimethylsiloxane (PDMS)-based microfluidic chip due to its low cytotoxicity and cost-effectiveness. With a quick turnaround time of 24 hours, the assay allows screening of three conditions for every 2 × 10^5^ cell under the current conditions. The comparative analysis demonstrated the superior performance of this platform compared to existing assays, offering flexibility in terms of the number of conditions screened based on throughput requirements and sample input size. It is possible to record single-cell drug responses using the readout method, allowing the identification and quantification of differences without compromising population statistics.

An integrated single-cell microfluidic drug screening platform, enhanced by droplet microfluidics, is presented as a transformative approach to overcome the limitations of traditional drug screening methods. As a result of its application to CML, it has the potential to provide targeted and effective cancer drug screenings.

## 2. Materials and methods

### 2.1 Cell culture

The leukemia cell lines K562 and NB4 were used as suspended cell lines and exhibited sensitivity and resistance to imatinib, respectively. These cell lines were procured from the Pasteur Institute and cultured in RPMI 1640 medium supplemented with 10% (v/v) fetal bovine serum (FBS) and 1% (v/v) penicillin–streptomycin under a 95% humidified atmosphere containing 5% CO_2_ at 37°C (Memmert, Germany). Cells were passaged every 2–3 days to ensure that the cells were in the exponential growth phase. Imatinib mesylate was generously provided by Merck & Co., Inc. (Linden, NJ, USA) and prepared according to the manufacturer’s instructions. Cell lines were treated with different concentrations of drug.

### 2.2 MTT assay

MTT colorimetric method was used to assess the viability of cells at different concentrations of imatinib. Briefly, K562 and NB4 cells were seeded at a density of 20 × 10^3^ cells per well in a 96-well plate after exposure to specified concentrations of imatinib (100 nM, 200 nM, 400 nM, 600 nM, 800 nM, and 1000 nM) for 24 or 48 hours. The cells treated with 10% DMSO were used as the positive control for cell death [16]. The cells were exposed to MTT solution (Sigma-Aldrich, St. Louis, MO, USA (0.5 mg/mL)) for 4 hours at 37°C and 5% CO_2_, after which the resulting formazan was dissolved in dimethyl sulfoxide (100 μL). The absorbance at 570 nm in each well was subsequently measured using an enzyme-linked immunosorbent assay reader.

### 2.3 Cell apoptosis assay

The impact of imatinib on the apoptosis of K562 and NB4 cells was assessed using an Annexin V/PI apoptosis kit according to the manufacturer’s instructions (BD Biosciences, CA, USA). Briefly, K562 cells were seeded at a density of 3 × 10^5^ cells per well in a 24-well culture plate. Following treatment with imatinib for 24 and 48 hours (200 nM, 400 nM, 600 nM, 800 nM), cells washed with cold PBS and stained with Annexin V-PI detection kit (BD Biosciences) according to the manufacturer’s instruction. Cells were then analyzed using flow cytometer (CyFlow@Space, sysmex, Foreningsgatan, Landskorna). Annexin V-positive and PI-negative cells were categorized as being in the early apoptotic phase, while cells with positive staining for both Annexin V and PI were considered to be undergoing late apoptosis. Our investigation determined that the most effective dose for treating K562 cells with imatinib is 600 nM, based on its IC50 dose. For subsequent drug screening, three concentrations were selected: 400 nM, 600 nM, and 800 nM.

### 2.4 Microfluidic drug screening device

The microfluidic device used in this study was designed using AutoCAD and simulated with COMSOL software to visualize fluid dynamics and droplet formation. Fabricated using soft lithography, a silicon wafer coated with SU-8 photoresist was patterned via UV exposure, and PDMS was cast to create microchannels that were bonded to a glass slide using oxygen plasma. To enhance droplet formation, the microchannels were treated with Aquapel to render them hydrophobic. The device featured two input channels: one for the aqueous phase containing the cell suspension and another for the oil phase, with droplets generated at a flow-focusing junction at a rate of 380 droplets per second, each approximately 60 μm in diameter. The device included four individual incubating chambers where single-cell droplets underwent drug screening, allowing for the simultaneous comparison of multiple conditions. The layout was designed to ensure that droplets circulated through all four chambers during the screening process, enhancing both the platform’s flexibility and efficiency in drug testing.

### 2.5 On-chip drug screening assay

To evaluate the effectiveness of the drug on K562 and NB4 cell lines at three chosen doses, four devices were fabricated using a mold designed according to the specifications mentioned earlier. In this process, one device was designated for screening a 400 nM imatinib dose for the K562 cell line, another for screening a 400 nM dose for the NB4 cell line, and the third and fourth devices for K562 without treatment (Control) and NB4 without treatment (Control), respectively. The resulting Annexin and PI staining data were compared with the Annexin and PI staining data obtained from cells in 24-well and 96-well plates. The comparison considered the duration of cell treatment and the percentage of apoptotic cells.

The study combined NB4 and K562 cell lines’ drug screening results to assess apoptosis levels in imatinib-treated K562 cells, ensuring the device’s effectiveness in apoptosis assessment.

The PDMS-based microfluidic chip previously mentioned was used for the drug screening process. In brief, the cells were aliquoted into 0.5 mL PCR tubes at final concentrations ranging from 1–2 ×10^6^ cells/mL. Then, 500 μL of the cell‒drug mixture, containing 2 × 10^5^ cells, was injected into the tube and separated by the oil phase at a 154.54 μL/h syringe pump withdrawal rate. This step was repeated for every 3-drug concentration for both cell lines and for control cells (K562 and NB4 cells without drug treatment) to compare the cell apoptosis rate and time measurements in a microfluidically designed device with a microtiter plate (98-well and 24-well plates). Untreated cells were used as a negative control. After loading each tube separately, the tubes were placed within the microfluidic chip, which had previously been back-flushed using an oil phase at a rate of 3245.45 μl/h via a syringe pump. After that, a syringe pump was utilized to infuse the combinations at a rate of 25 μL/h. Ultimately, the tubing at the inlet and outlet was cut and wrapped with parafilm. The chips were placed in a 150 mm cell culture dish containing wet paper towels and transferred to a humidified incubator at 37°C supplemented with 5% CO2 for 24–48 h of incubation. Fluorescence images (Ex. 531/40 nm, Em. 593/40 nm) were taken at 10x magnification to confirm the generation of droplets. Fluorescence images are a powerful tool for confirming droplet generation in microfluidic systems. They provide a visual confirmation of the presence, size, and distribution of droplets, offering insights into the success and uniformity of the emulsification process.

After the 24- and 48-hour incubation periods, the devices were removed from the incubator, and upon injecting a specified amount of culture medium, the incubated droplets were collected. After merging the collected droplets, according to the instructions for Annexin and PI staining, the cells were harvested, washed with cold PBS, and resuspended in 100 μL of reaction buffer containing 5 μL of Annexin V–fluorescein isothiocyanate (FITC) and 5 μL of PI. Following a 15-minute incubation at room temperature in the dark, 400 μL of binding buffer was added to each sample. Fluorescence-activated cell sorting (FACS; BD Biosciences, San Jose, California, USA) was then employed to analyze the stained cells. Annexin V-positive and PI-negative cells were classified as being in the early apoptotic phase, while cells displaying positive staining for both Annexin V and PI were identified as undergoing late apoptosis.

### 2.6 Microtiter plate drug screening assay

Drug screening assays were conducted in clear, round, flat-bottom 24-well and 96-well microtiter plates. Cells were seeded at densities of 3.0 × 10^5^ cells per well for 24-well plates and 5.0 × 10^4^ cells per well for 96-well plates. The drugs were diluted in culture media before being added, resulting in the final drug concentrations (400 nM, 600 nM, or 800 nM) shown on the graphs. Along with the devices used for placement, the plates were incubated in a humidified environment at 37°C with 5% CO2 supplementation for a 24–48-hour period. Subsequently, Annexin/PI was utilized to evaluate cell viability. The negative controls included cells treated without the drug, while the blank controls included wells with no cells. All experiments were conducted in triplicate for microfluidic devices and in both 24- and 96-well plates.

### 2.7 Statistical analysis

The dose effect of imatinib treatment was analyzed using two-way ANOVA using GraphPad Prism v10.1.0.316.

## 3. Results

### 3.1 Microfluidic chip design and fabrication

**Fig 1:**
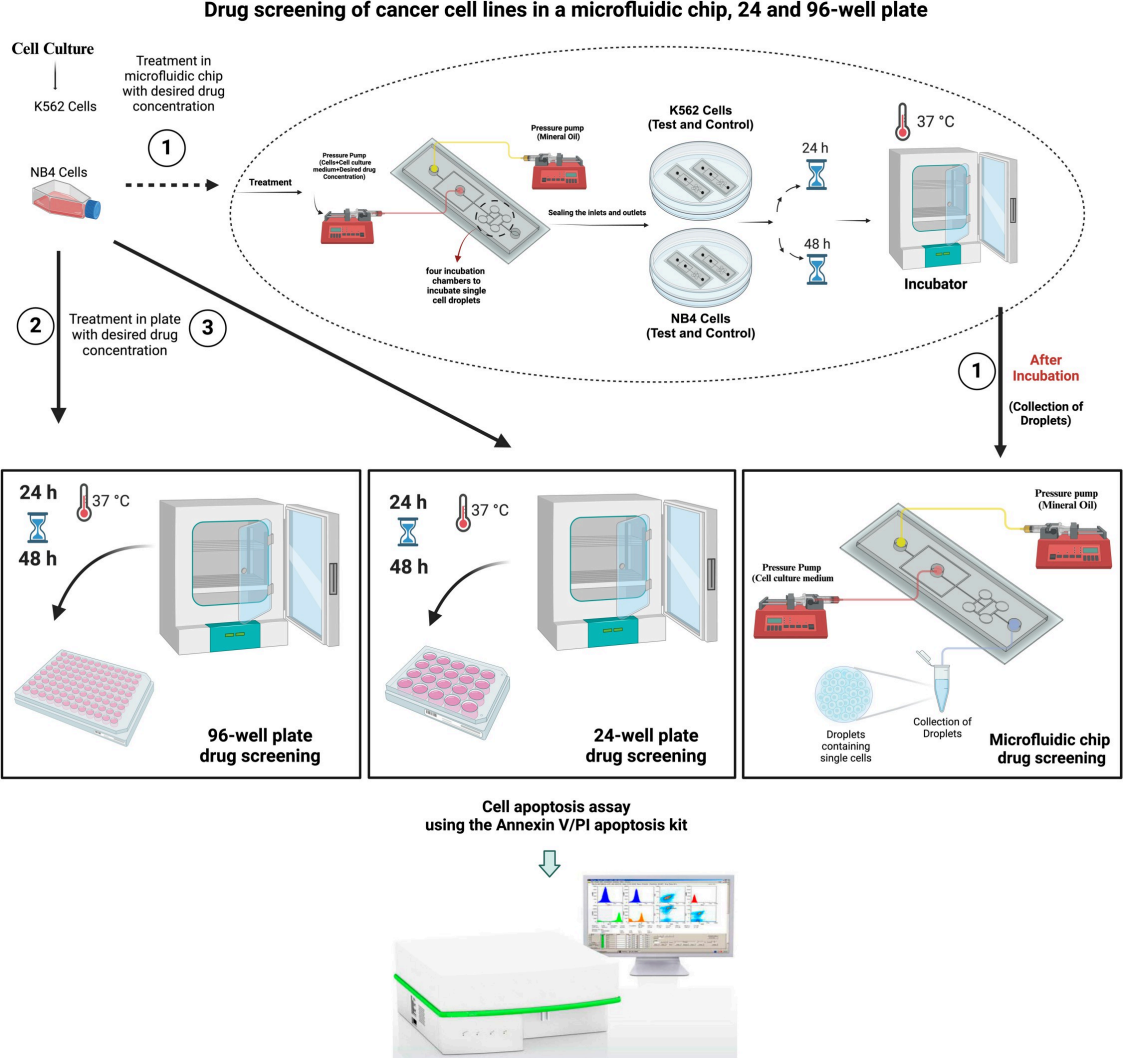
On-chip drug screening assay evaluating the efficacy of imatinib at various concentrations on K562 and NB4 cells (400 nM, 600 nM, and 800 nM) versus traditional drug screening in 24-well and 96-well plates. The analysis included cell treatment duration and apoptosis percentage assessment for both the K562 and NB4 cell lines. (Created in BioRender. Jaafari, N. (2024) https://BioRender.com/i83c416).

**Fig 2:**
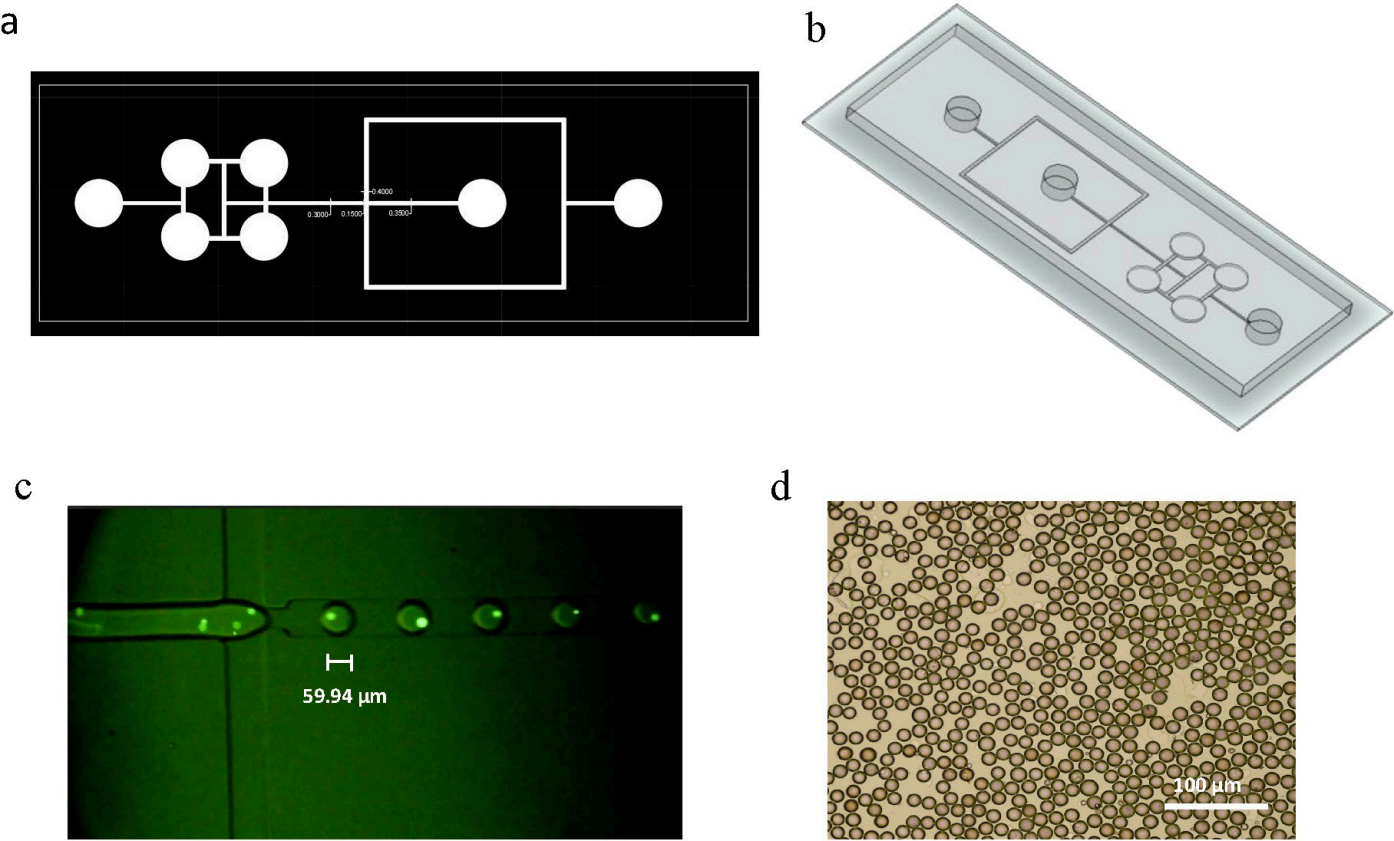
Advanced visualization techniques in on-chip drug screening. a, b) Visual representation created with AutoCAD and SolidWorks elucidates the on-chip drug screening assay method outlined in the article, c) Characterization of microdroplet generation in the flow-focusing chip using fluorescent cell staining dye (CFSE) captured by a high-speed CMOS, fast camera Nikon D5300 and d) Visualization of droplets through an inverted microscope.

**Fig 3:**
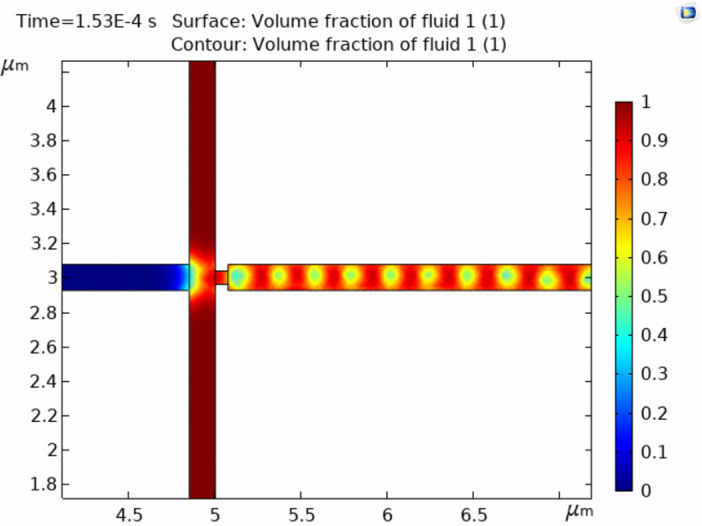
COMSOL simulation of droplet formation by the designed microfluidic device.

Fig 1 shows a schematic illustration of the on-chip drug screening test. In brief, the microchannel architectures were created using AutoCAD 2019 software (Autodesk, Inc., San Rafael, CA, USA) (Fig 2), and COMSOL Software for Multiphysics simulation, which depicted the dynamic process of droplet formation within the designed microfluidic device. The simulation captures intricate fluid dynamics, highlighting key parameters influencing droplet generation, such as flow rates, channel geometry, and surface tension. The visual representation offers insights into the device’s functionality and performance in generating uniform and controlled droplets (Fig 3).

The microfluidic device was fabricated using traditional soft lithography techniques. A silicon wafer was coated with SU-8 2050 photoresist (Microchem, USA) and patterned using UV exposure and a photolithography mask. The PDMS mixture (Sylgard 184; Dow Silicones, Midland, MI, USA), with a 15:1 w/w ratio of elastomer to curing agent, was poured into the SU-8-on-Si wafer master, vacuumed for 20 minutes to remove air bubbles, and cured at 65°C for 4 hours in a laboratory oven. Following curing, the PDMS was carefully peeled away from the master and trimmed to size. Holes for the inlet and outlet were punched using a 1 mm biopsy punch (KAI, Japan). The PDMS was bonded to a glass slide (Electro Technic Products, Chicago, IL) using oxygen plasma. The microchannels were rendered hydrophobic using Aquapel (PPG Industries, USA) to enhance droplet generation. Sterilization was achieved with 70% ethanol and UV exposure. The microchannels had a height of 75 μm, with inlet and outlet channel widths of 150 μm.

#### 3.1.1 Droplet formation and flow control in comparison

**Fig 4:**
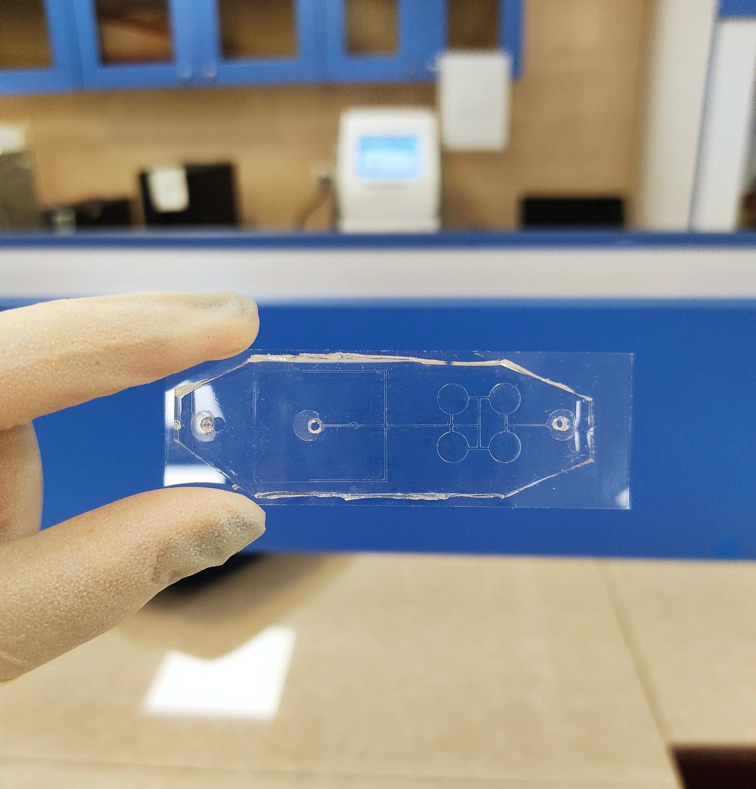
The actual image of the designed device.

The aqueous phase (dispersed phase), containing the cell suspension, is introduced through one input channel, while the oil phase (continuous phase), composed of mineral oil with 2% stabilizing surfactants (Abil EM 180, Degussa/Goldschmid), surrounds the aqueous droplets, preventing coalescence and ensuring droplet stability. The interaction between the two phases at the flow-focusing junction leads to the controlled generation of uniform droplets. The aqueous and oil flow rates were set at 154.54 μl/h and 3245.45 μl/h, respectively (a ratio of 1:21), using two syringe pumps (Harvard PHD ULTRA and New Era NE-8000). Droplets with a diameter of 59.94 μm and a volume of 8.5 nl were produced at a generation rate of 380.69 Hz. Droplet formation was observed using a Nikon inverted microscope and captured with a high-speed, quick-focusing CMOS camera (Nikon D5300) (Fig 4).

This droplet-based system enables precise control over droplet formation and size, allowing for high-throughput, single-cell drug screening. The novelty of the system lies in its ability to generate reproducible and stable droplets by combining the aqueous (dispersed) and oil (continuous) phases in a flow-focusing microfluidic chip. This platform significantly reduces screening time while maintaining accuracy, making it a powerful tool for drug testing.

The device features four individual incubating chambers where single-cell droplets undergo drug screening assessments. These chambers allow for the comparison of multiple conditions simultaneously. The layout is designed to ensure that droplets circulate through all four chambers during the drug screening process, enhancing the platform’s flexibility and efficiency.

### 3.2 Imatinib attenuates cell viability in a time- and dose-dependent manner

**Fig 5:**
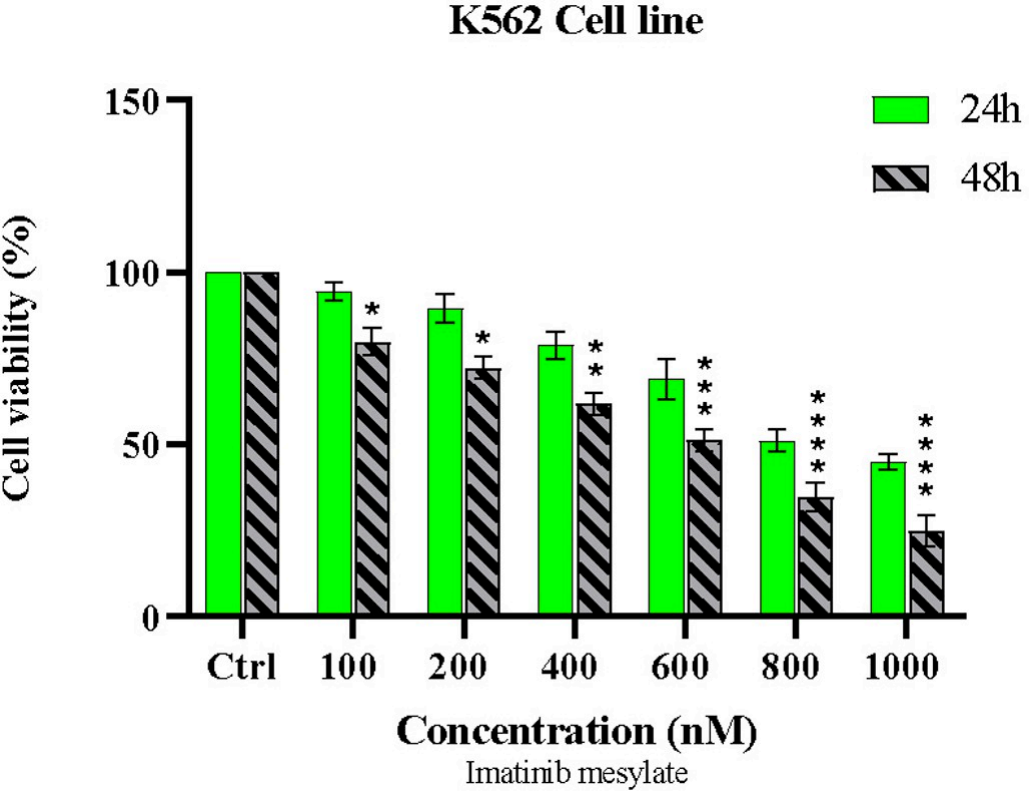
Imatinib induces dose-dependent in K562 cells. Cells were treated with escalating doses imatinib for 24 and 48 hr. Cell viability assessed using MTT assay. (Plot: Mean with SD, n = 3, *p<0.05, **P < 0.01, ***P <0.001).

**Fig 6:**
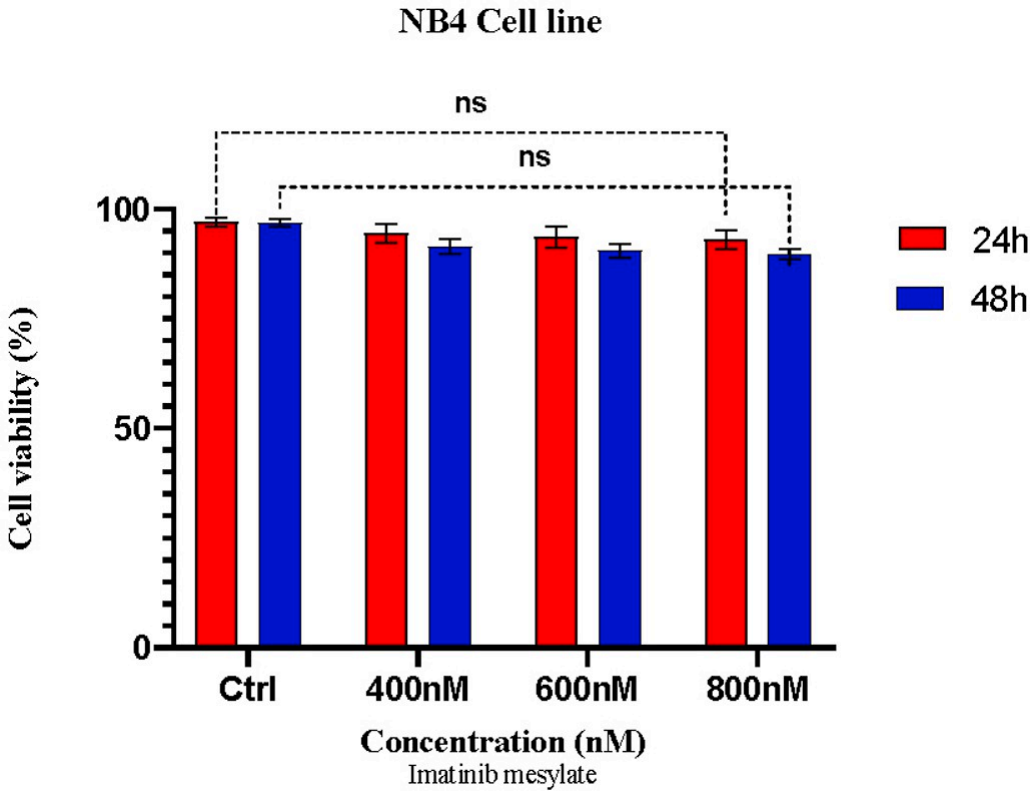
The effect of imatinib on the viability of NB4 cells. NB4 cells were treated with three distinct concentrations of imatinib for 24 and 48 hr. The viability was assessed using MTT method. (Plot: Mean with SD, n = 3, *p<0.05, **P < 0.01, ***P < 0.001).

Following imatinib treatment, the viability of K562 and NB4 cells was evaluated using the MTT assay. Fig 5 shows that imatinib treatment causes dose- and time-dependent cell death. IC50 values were 850 nM and 640 nM after 24 and 48 hours of drug treatment in 24 well plate, respectively. Based on a one-way analysis of variance, we confirmed that K562 cells were significantly less viable 48 hours after exposure to 600 nM imatinib compared to 24 hours later. As expected, imatinib has no effect on NB4 cells (Fig 6).

To confirm the results obtained from the MTT assay for determining the optimal concentration of imatinib in subsequent drug screening analyses, an apoptosis assay was conducted using flow cytometry (Figures will be provided upon request).

As shown in Fig 6, treatment with 600 nM imatinib had no significant effect on the NB4 cell line, used as negative control, confirming its inherent resistance to the drug. In contrast, K562 cells, which are sensitive to imatinib, exhibited a substantial reduction in cell viability at the same concentration. Although the IC50 value of 600 nM is specific to the K562 cell line, we applied this concentration to NB4 cells for comparative purposes. By maintaining consistent experimental conditions across both cell lines, we aimed to highlight the stark difference in drug response between imatinib-sensitive K562 cells and imatinib-resistant NB4 cells. The absence of a response at 600 nM further supports their role as a negative control in the experiment. This consistent concentration enables us to draw meaningful conclusions about the drug screening performance of our microfluidic platform across cell lines with differing drug sensitivities.

### 3.3 The microfluidic device enhances imatinib efficacy and reduces the screening time to 24 hours

**Fig 7:**
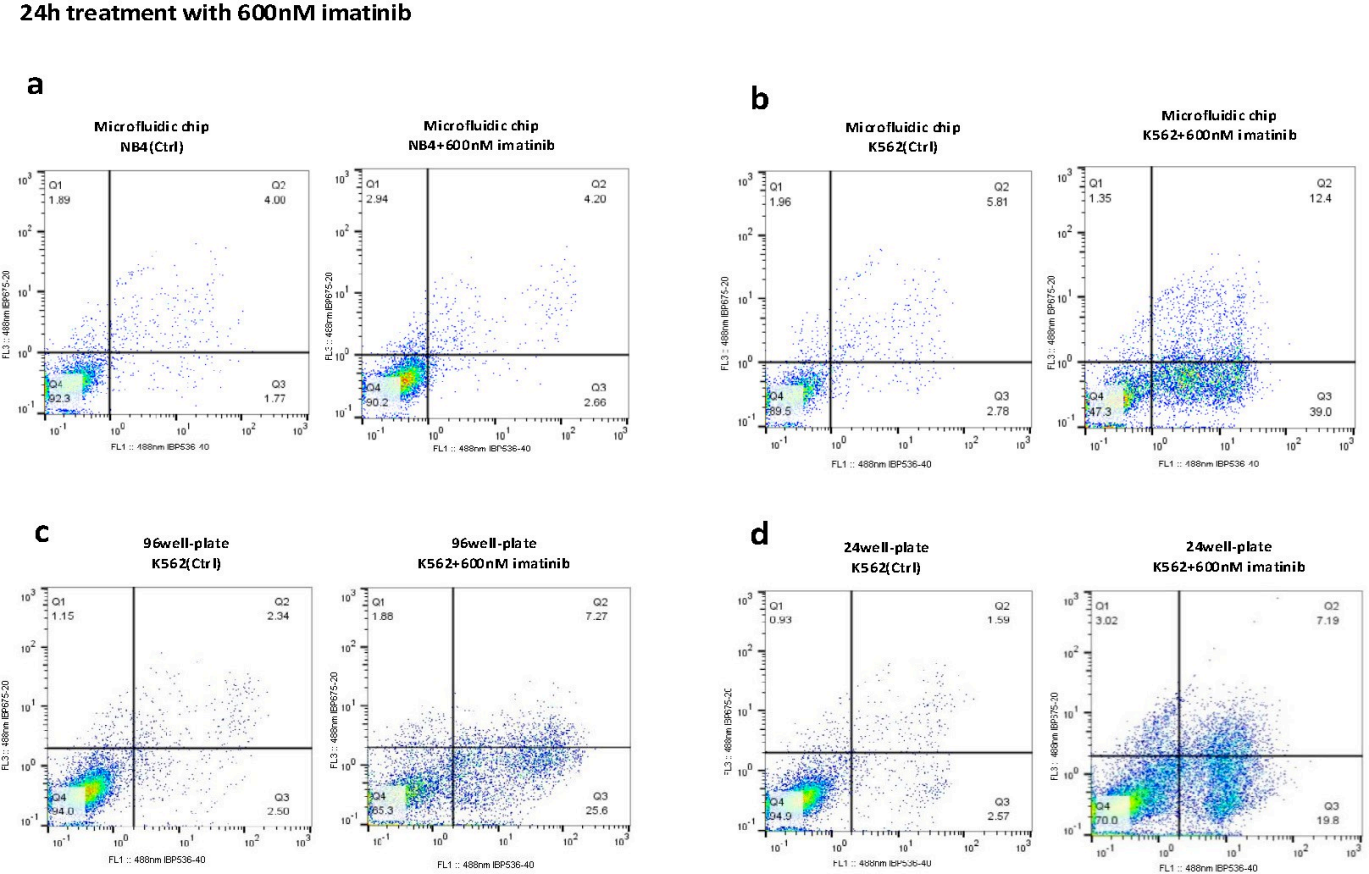
Comparative flow cytometric analysis of 24h imatinib-induced apoptosis in K562 and NB4 cells. Flow cytometric analysis was performed as follow: a) NB4 cells treated with 600 nM imatinib in a microfluidic chip, b) K562 cells treated with 600 nM imatinib in a microfluidic chip, c) K562 cells treated with 600 nM imatinib in a 96-well culture plate, d) K562 cells treated with 600 nM imatinib in a 24-well culture plate for 24 hours. The histograms represent necrotic (Q1), late apoptotic (Q2), early apoptotic (Q3), and viable cells (Q4). The percentage of total apoptotic cells was calculated from Q2 + Q3. The figure provides a visual representation of the differential apoptotic response across different culture conditions and treatment durations.

**Fig 8:**
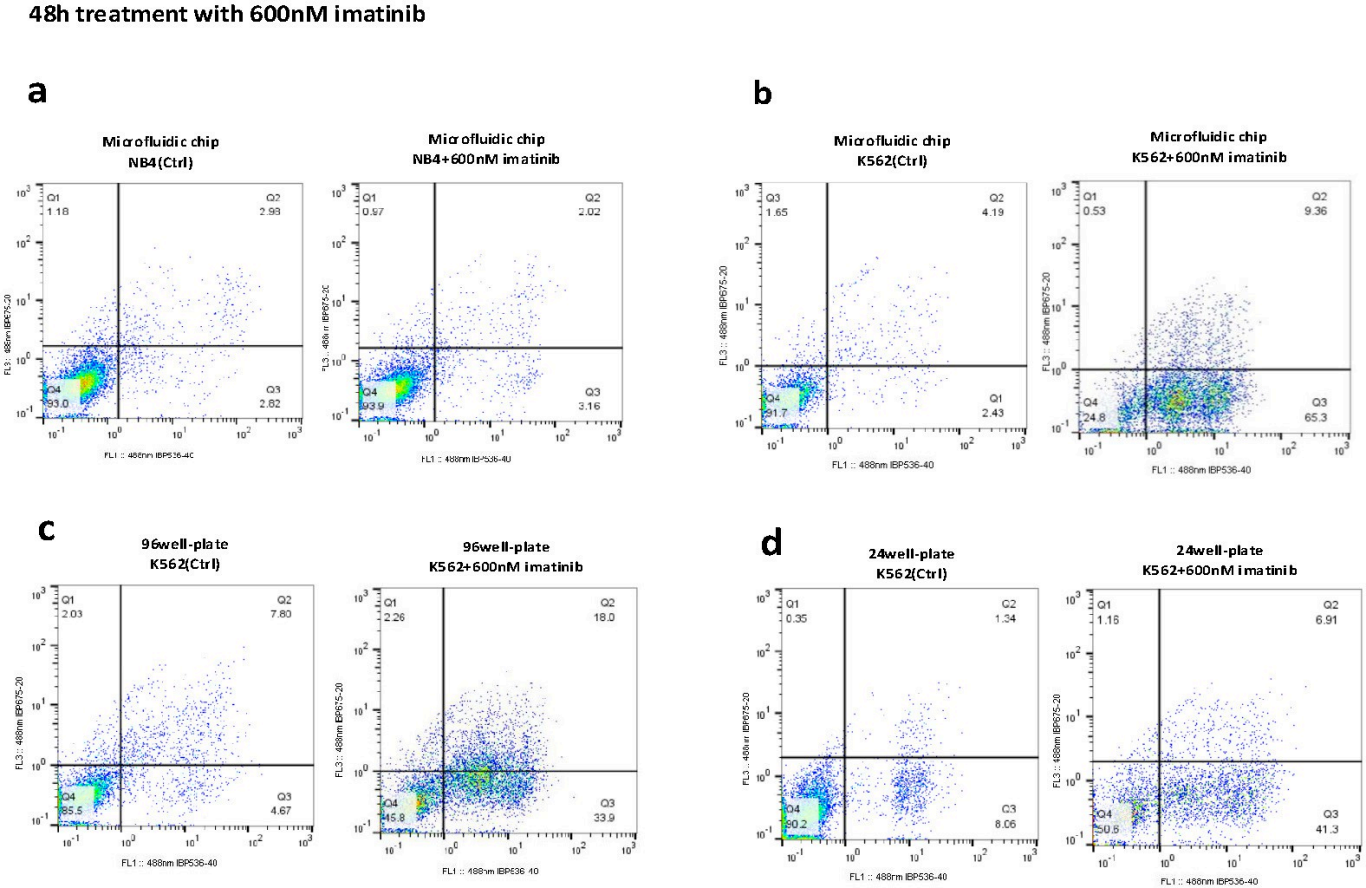
Comparative flow cytometric analysis of 48h imatinib-induced apoptosis in K562 and NB4 cells. Flow cytometric analysis was performed as follow: a) NB4 cells treated with 600 nM imatinib in a microfluidic chip, b) K562 cells treated with 600 nM imatinib in a microfluidic chip, c) K562 cells treated with 600 nM imatinib in a 24-well culture plate, d) K562 cells treated with 600 nM imatinib in a 96-well culture plate for 48 hours. The histograms represent necrotic (Q1), late apoptotic (Q2), early apoptotic (Q3), and viable cells (Q4). The percentage of total apoptotic cells was calculated from Q2 + Q3. The figure provides a visual representation of the differential apoptotic response across different culture conditions and treatment durations.

**Fig 9:**
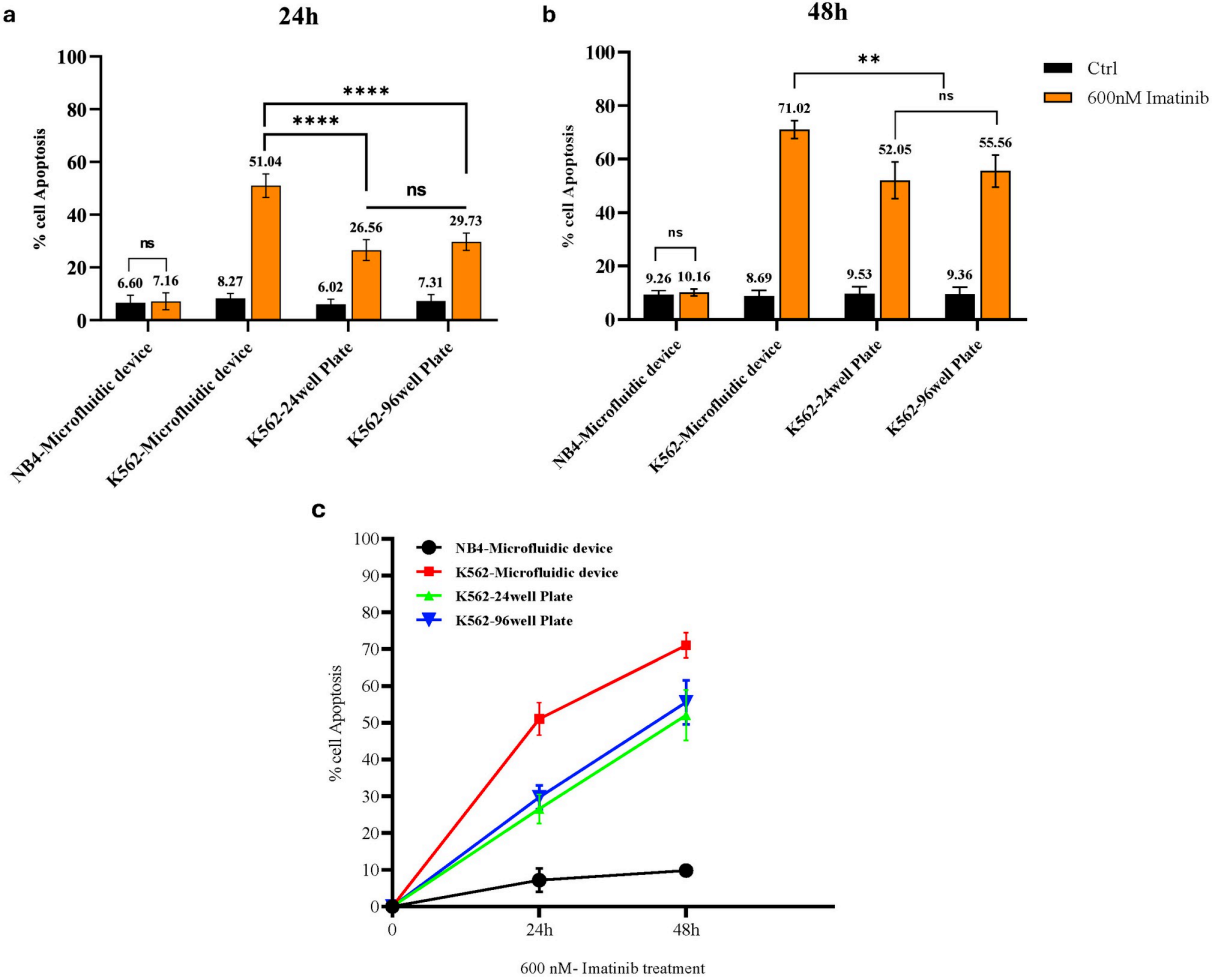
Comparative apoptosis analysis in NB4 and K562 cells across different culture platforms and imatinib treatment durations. a, b) Comparative analysis of apoptosis rates in NB4 and K562 cells cultured in microfluidic devices, 96-well culture plates, and 24-well plates, with or without 600 nM of imatinib treatment for 24 and 48 hours. c) K562 cells were treated with the imatinib concentration of 600 nm in different culture platforms at 24 and 48 hours. Notably, the use of this microfluidic technology considerably reduced the duration of drug screening from 48 hours to a more efficient 24 hours.

As mentioned before, drug screening was conducted on K562 and NB4 cells using a microfluidic device, as well as 24- and 96-well plates. Cells were treated with imatinib at concentrations of 400 nM, 600 nM, and 800 nM. Apoptosis rates were measured at 24- and 48-hours post-treatment using an Annexin-PI detection kit. The results showed that the apoptotic rate of K562 cells subjected to 24-hour imatinib treatment within the microfluidic device was significantly greater than that of K562 cells cultured in 24- and 96-well plates, with rates of 51.4% in the microfluidic device compared to 26.99% in 24-well plates and 32.87% in 96-well plates, calculated from Q2 + Q3 as shown in Fig 7B–7D (statistical analysis reported in Fig 9 (p<0.0001)). As depicted in Fig 8B–8D, the apoptotic rate of K562 cells was significantly elevated after 48-hour imatinib treatment in the microfluidic device, with rates of 74.66% compared to 48.21% in 24-well plates and 51.9% in 96-well plates, calculated from Q2 + Q3 (p<0.001) (Fig 9). Notably, the apoptotic rate of untreated K562 cells within the microfluidic device did not significantly differ from that of the corresponding untreated cell population in 24- or 96-well plates at either 24 or 48 h post treatment (p >0.05) (Fig 9). The enhanced efficacy observed in the microfluidic device is due to the controlled microenvironment that improves the interaction between the drug and the cells, rather than being an effect of the imatinib drug or the device alone. The results of the same experiment with the other doses are shown in S1 and S2 Figs.

Furthermore, comparing untreated K562 and NB4 cells, which were cultured in the microfluidic device and treated with imatinib, NB4 cells did not exhibit a significant increase in the percentage of apoptotic cells (p >0.05) (Fig 9), confirming the resistance of these cells to imatinib and rejecting the effect of the device on cell apoptosis. Importantly, there was no notable difference in apoptosis or overall cell death rate between NB4 cells treated with imatinib in the microfluidic device and those treated in 24- or 96-well culture plates (p >0.05) (Fig 9), further confirming that the cellular culture conditions in the microfluidic device did not induce any apoptotic or necrotic stress in the cells.

Moreover, concerning the duration of exposure, imatinib treatment at a concentration of 600 nM resulted in about 50% apoptosis following 48 hours of treatment in conventional culture plates. The use of a microfluidic device with the same imatinib concentration led to a similar 50% apoptosis rate within a shortened 24-hour treatment period. Notably, this microfluidic device significantly reduced the duration of drug screening from 48 hours to 24 hours (Fig 9C).

## 4. Discussion

In this study, we used a 5 × 2.5 cm microfluidic chip made from PDMS for droplet microfluidics-based drug screening of the K562 cancer cell line. PDMS is widely known for its low cytotoxicity and cost-effective fabrication, making it suitable for clinical applications. This platform enables the screening of three conditions for every 2 × 10^5^ cell with a rapid 24-hour turnaround time. Additionally, our method allows the assessment of single-cell drug responses, while maintaining population-level analysis using multiple droplets for each drug condition. Droplet microfluidics focuses on generating and controlling individual droplets in microchannels using immiscible multiphase flows [17, 18]. Each droplet acts as a small independent microreactor that can be transported, mixed, and analyzed separately. This approach significantly boosts throughput and scalability without adding complexity to the device [1, 18–20]. This technology has been applied in diverse fields such as material synthesis, biochemical analysis, and biomedical research [21]. In drug discovery, techniques like high-throughput screening, drug concentration generators, 3D cell cultures, and single-cell analysis have improved drug screening efficiency [22–24]. Due to cell heterogeneity, individual cells often show different responses to drugs, making single-cell screening essential for understanding these varied reactions [6, 25]. Brouzes et al. developed a droplet microfluidic platform for high-throughput single-cell drug screening [26]. They emulsified drug libraries with fluorescent labels and combined them with droplets containing individual cells. After 24 hours of incubation, fluorescence analysis was used to assess cell viability, enabling rapid evaluation of drug effects at different concentrations. To improve the accuracy of viability assessments and minimize potential biases introduced by fluorescent labeling, we used Annexin V and propidium iodide (PI) staining after the incubation period. This allowed us to examine apoptosis over a longer duration and gave a clearer understanding of how drugs impact cellular responses.

Clausell-Tormos et al. developed an on-chip high-throughput screening system by integrating an autosampler and a tree-shaped splitter, increasing the screening capacity to 100 conditions in an enzyme inhibition assay [27]. Fluid samples with varying inhibitor concentrations were extracted from a 96-well plate and divided into three streams for simultaneous processing. These were then combined with the enzyme and its fluorogenic substrate. Similarly, Bithi and Vanapalli introduced an innovative microfluidic technology for drug testing, focusing on isolating single tumor cells and their clusters [28]. This method uses the precise control of fluid volumes to create droplets that encapsulate individual cell. The platform allows for the analysis of drug responses with high precision and efficiency. These droplets enable the precise analysis of drug effects on tumor cells, offering valuable insights into the efficacy and side effects of pharmaceutical compounds, which is critical for developing personalized cancer treatments. Wong’s research highlights the use of droplet microfluidics for drug screening on cancer cell lines and primary tumors [29]. This method enhances both the efficiency and precision of drug screening, allowing for more accurate results in less time. Although combining multiple analytical techniques in microfluidic systems can improve functionality, it can also create challenges related to compatibility, calibration, and standardization. To overcome this, we designed a simplified droplet generation process that avoids the need for complex calibration [26]. Our microfluidic chip features channels that are 75 micrometers in height, with inlet and outlet diameters of 5 millimeters. It has two input channels: one for the aqueous phase and another for the oil phase, used to generate encapsulated droplets. The chip also includes four separate incubation chambers (each 5 millimeters in diameter), where single-cell droplets undergo drug screening. Each chamber is connected to the next, allowing for continuous circulation of droplets. This design simplifies the experimental process, reduces technical challenges, and enhances the robustness and usability of the platform for drug screening. Additionally, it improves screening accuracy and enables dynamic exploration of drug responses across different cell types and tumor samples. S.-K. Zhao and collaborators introduced a rapid on-chip drug screening method using acoustic streaming technology [16]. This innovative approach significantly reduces screening time compared to traditional methods. By using acoustic waves to move fluids within microchannels, this setup allows for the fast manipulation and screening of leukemia cells under different drug conditions. With this technique, drugs can impact cell viability in less than 30 minutes, compared to the typical 24 hours in standard methods. This acoustic-based drug delivery approach saves both time and costs when used with microfluidic systems for multi-concentration drug screening [30, 31].

Our device, with its specialized design, reduced the drug screening time from 48 hours to 24 hours. The shortened screening time results from the precise control over fluid flow and enhanced drug-cell interaction in the microscale environment. Real-time observation capabilities further speed up the process by enabling quick assessments of cellular responses, helping researchers identify potential drug candidates more efficiently. Our results showed that the chip assay effectively captures variations in drug responses in leukemic cell lines, making it a valuable tool for evaluating different treatments. By analyzing the average cell viability data, we can rank drug effectiveness within a panel. Additionally, examining the range of droplet viability provides insights into the consistency of drug responses, offering a broader view of cellular outcomes. While our study primarily focuses on suspended leukemia cell lines, we recognize the importance of extending the application of our system to solid tumor cells, which are typically adherent. Solid tumor cells present different challenges compared to suspended cell lines due to their need for surface attachment in order to proliferate and maintain their functional characteristics. However, several modifications can be made to the PDMS surface to enhance its ability to support solid tumor cell attachment and growth. One strategy involves coating the PDMS surface with extracellular matrix (ECM) proteins such as collagen, fibronectin, or laminin, which mimic the natural environment of solid tumor cells and provide the necessary biochemical signals for attachment and proliferation. Another approach is to incorporate hydrogels like Matrigel into the microfluidic system. Hydrogels offer both structural and biochemical support, creating a 3D microenvironment that closely mimics the in vivo conditions of solid tumors, thereby promoting cell attachment and growth. Additionally, adherent cells can be cultured using microcarrier beads, which offer a solid surface for cell attachment while still allowing the cells to remain suspended in the droplet system. These approaches have been successfully applied in other microfluidic systems and could be adapted for our platform to support the growth of adherent solid tumor cells.

Our method’s flexibility in adjusting cell concentrations and drug volumes has proven effective for rapid, low-input drug screening of leukemic cells. For a thorough evaluation of drug potency across a panel, however, a single platform quantitative assessment is recommended to minimize system errors and assay variations. It is important to recognize that in vitro assays do not account for the pharmacokinetic differences seen in vivo, so these results should be combined with clinical expertise to guide therapeutic decisions.

## 5. Conclusion

In conclusion, this study demonstrates that the microfluidic platform offers a more efficient method for drug screening compared to traditional 24- and 96-well plates. The platform reduces drug screening time from 48 hours to 24 hours, highlighting its potential to improve the efficiency of drug screening processes.

## Supporting information

S1 Fig Comparative flow cytometric analysis of 24h and 48h 400 nM imatinib-induced apoptosis in K562 and NB4 cells. (TIFF)

S2 Fig Comparative flow cytometric analysis of 24h and 48h 800 nM imatinib-induced apoptosis in K562 and NB4 cells. (TIFF)
