# Supplementary figures and images for: Design and fabrication of novel microfluidic-based droplets for drug screening on a chronic myeloid leukemia cell line
Source: PLoS One. 2025 Jan 15;20(1):e0315803. doi: 10.1371/journal.pone.0315803 (PMC11734902; doi:10.1371/journal.pone.0315803)

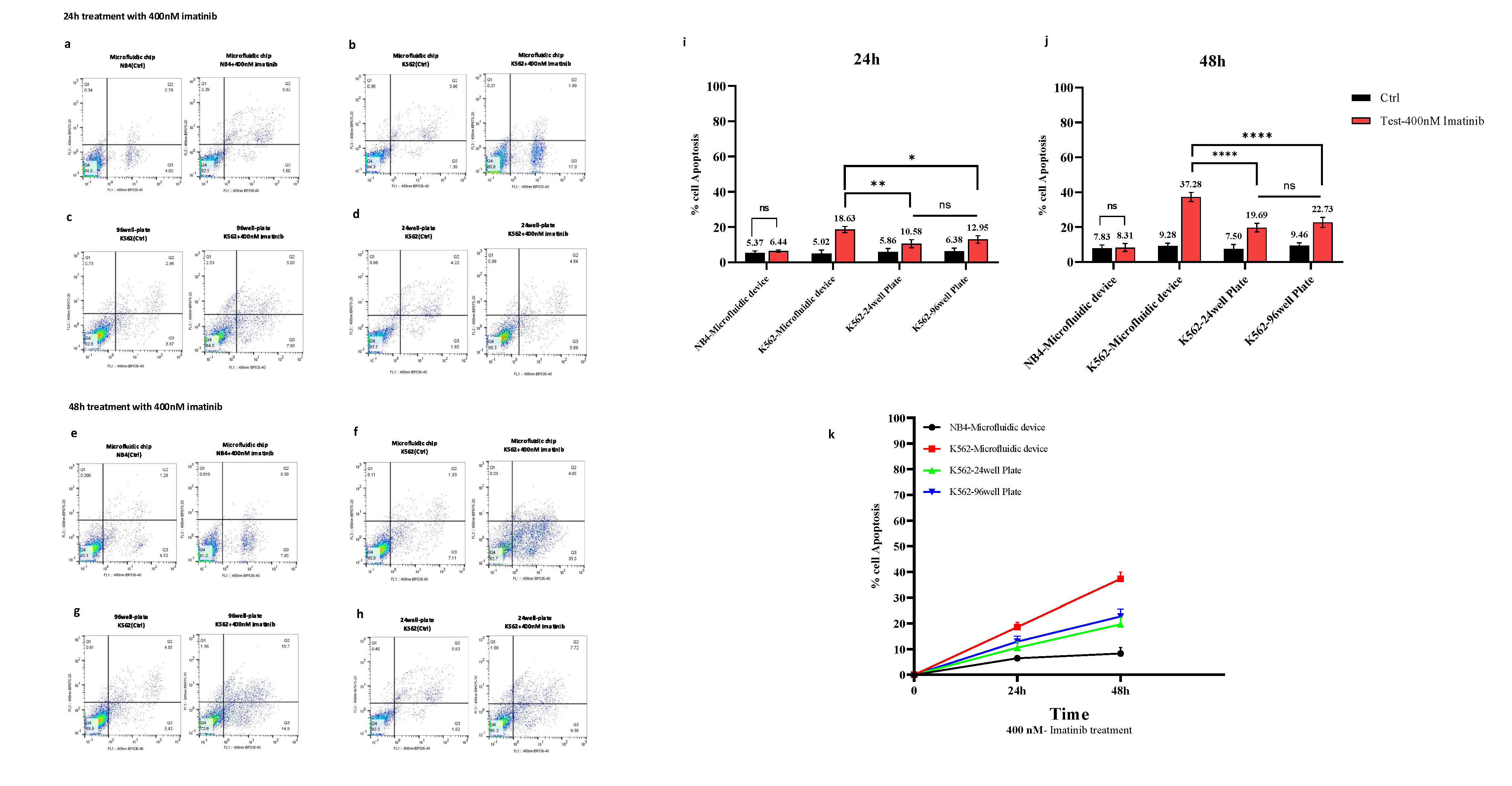

Supplement: S1 Fig — (TIFF) [file pone.0315803.s001.tiff]

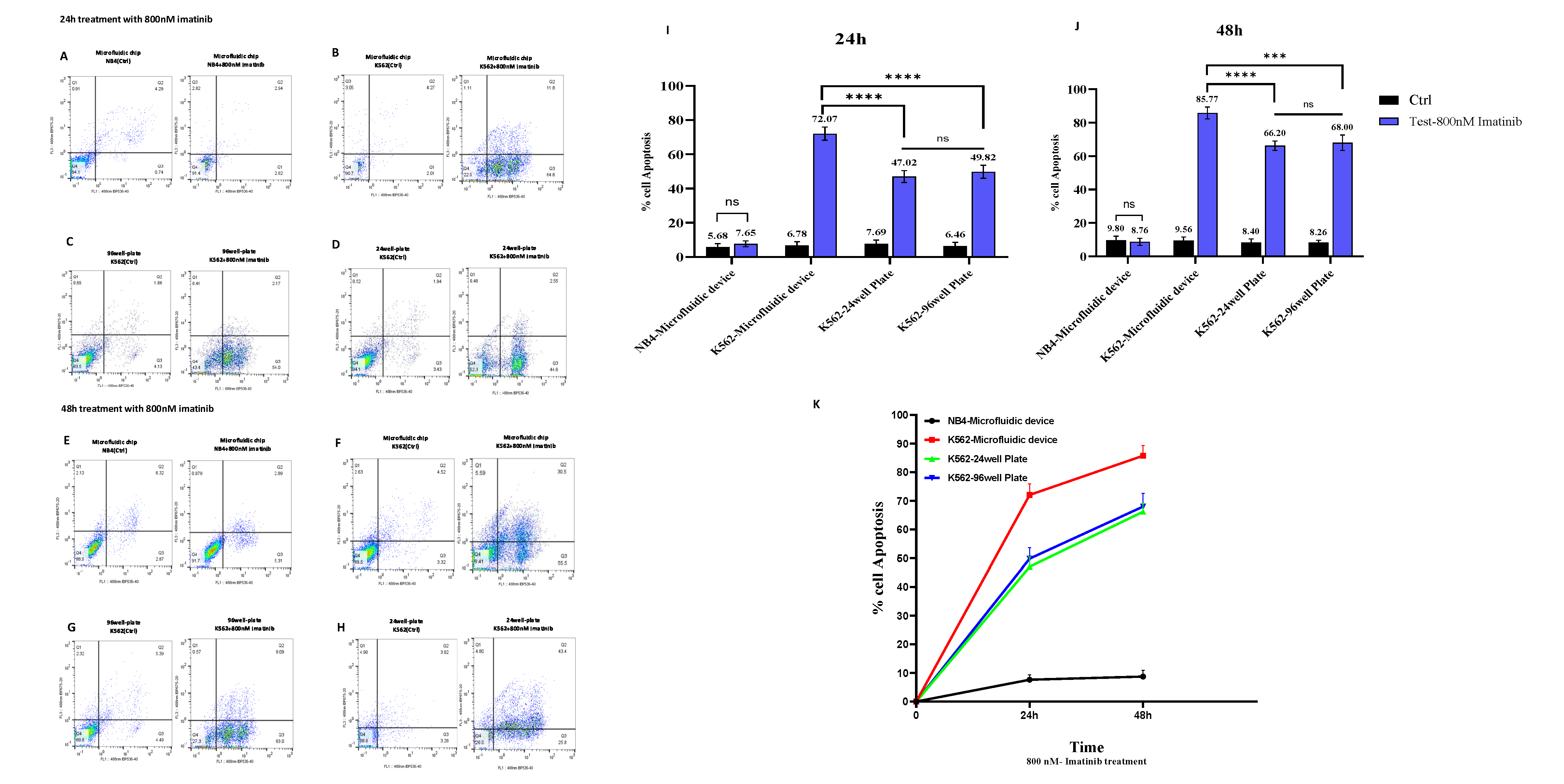

Supplement: S2 Fig — (TIFF) [file pone.0315803.s002.tiff]
